# A Bio-Electro-Fenton System Employing the Composite FePc/CNT/SS316 Cathode

**DOI:** 10.3390/ma10020169

**Published:** 2017-02-13

**Authors:** Yi-Ta Wang, Ruei-Shiang Wang

**Affiliations:** Department of Mechanical and Electro-Mechanical Engineering, National Ilan University, Yilan 26047, Taiwan; r0322009@ems.niu.edu.tw

**Keywords:** bio-electro-Fenton microbial fuel cells, iron phthalocyanine, carbon nanotubes

## Abstract

Bio-electro-Fenton microbial fuel cells generate energy through the decomposition of organic matter by microorganisms. The generated electricity drives a Fenton reaction in a cathode chamber, which can be used for the decolorization of dye wastewater. Most of the previous works added expensive platinum catalyst to improve the electrical property of the system. In this research, aligned carbon nanotubes (CNTs) were generated on the surface of SS316 stainless steel by chemical vapor deposition, and an iron phthalocyanine (FePc) catalyst was added to fabricate a compound (FePc/CNT/SS316) that was applied to the cathode electrode of the fuel cell system. This was expected to improve the overall electricity generation efficiency and extent of decolorization of the system. The results showed that the maximum current density of the system with the modified electrode was 3206.30 mA/m^2^, and the maximum power was 726.55 mW/m^2^, which were increased by 937 and 2594 times, respectively, compared to the current and power densities of a system where only the SS316 stainless steel electrode was used. In addition, the decolorization of RB5 dye reached 84.6% within 12 h. Measurements of the electrical properties of bio-electro-Fenton microbial fuel cells and dye decolorization experiments with the FePc/CNT/SS316 electrode showed good results.

## 1. Introduction

A microbial fuel cell is a cell module that converts chemical energy to electrical energy with the help of microbial strains [1]. A typical double-chamber microbial fuel cell contains an anode chamber and a cathode chamber, and employs a proton exchange membrane between the anode and cathode for separation. Air flowing into the cathode chamber is blocked by this proton exchange membrane and does not affect the electricity generation of the microorganisms at the anode. The protons generated by the anode can be transmitted through the proton exchange membrane located in the middle to the cathode, and the cathode chamber can generate water by combining the electrons and protons transmitted from the external circuit with oxygen. The electrochemical reactions that occur during the operation of the microbial fuel cell are similar to those that occur in common methanol fuel cells, and are shown in Equations (1) and (2), with glucose as the nutrient [2]:

C_6_H_12_O_6_ + 6H_2_O → 6CO_2_ + 24H^+^ + 24e^−^, (1)

O_2_ + 4H^+^ + 4e^−^ → 2H_2_O, (2)

Bio-electro-Fenton microbial fuel cells integrate microbial fuel cell and E-Fenton technologies, where the anode chamber generates electrons and hydrogen ions by the decomposition of organic matter with microbial strains to drive the cathode Fenton reaction, thus degrading sewage and wastewater solutions that the microorganisms cannot degrade. The electron-Fenton reaction pathway is described in Equations (3)–(6):

Fe^2+^ + H_2_O_2_ → ·HO + OH^−^ Fe^2+^ + H_2_O_2_ → Fe^3+^ + ·HO + OH^−^, (3)

HO + Fe^2+^ → Fe^3+^ + OH^−^, (4)

Fe^3+^ + e^−^ → Fe^2+^, (5)

2H^+^ + O_2_ + 2e^−^ → H_2_O_2_, (6)

A study by Feng showed that the energy generated by the Shewanella anode of a microbial fuel cell enabled the cathode to generate ferrous ions and hydrogen peroxide. The generated hydroxyl radical degraded methyl orange dye, with 100% degradation being achieved within 14 h [3]. Fernández de Dios studied the decolorization of five different types of dye wastewater using a microbial fuel cell, and found that the decolorization percentages of four of the five types were greater than 88% [4]. Wang treated toxic sewage using bio-electro-Fenton microbial fuel cells that generated hydrogen peroxide and ferrous ions. The results showed that the highly toxic trivalent arsenic ion was converted into the pentavalent arsenic ion, which has lower toxicity [5]. Zhang applied bio-electro-Fenton microbial fuel cells to the macromolecular aqueous solutions generated during dye degradation and various pharmaceutical processes, and the degradation efficiency after 9 h was 70% [6]. This showed that bio-electro-Fenton microbial fuel cells do not need continuous additions of the Fenton reagent, and the iron source in the system has a self-sustaining mechanism, which is conducive to the decolorization and degradation of various types of dye wastewater, toxic waste solutions, and polymer solutions.

When selecting the electrode materials for bio-electro-Fenton microbial fuel cells, the anode materials should have (1) good conductivity and low impedance; (2) excellent biocompatibility; (3) stable chemical properties; (4) good corrosion resistance; and (5) good mechanical properties [7,8,9,10]. The cathode electrode needs to have good electrochemical properties because a strong oxidation-reduction ability may be conducive to electron transport. The best result is usually achieved when platinum is added as the catalyst. This is mainly because platinum can reduce the cathode activation energy and improve the reaction rate [11]. In Moon’s research, the density of the power generated by a graphite electrode coated with platinum was 150 mW/m^2^, which was three times the energy generated by a pure graphite electrode. This proved that platinum can improve the electrocatalytic activity and reduce the barrier caused by the activation of the over-potential [12]. However, platinum is a precious metal, which may increase the fabrication cost of a microbial fuel cell. Many studies have been conducted with the goal of reducing the proportion of platinum, or finding other catalysts as substitutes for platinum. Transition metallic macrocycles are often used as catalysts in studies on the cathode electrode of a microbial fuel cell because they have good oxidation-reduction abilities, which improve the efficiency and lower the cost compared to the use of platinum [13,14,15].

Macrocyclic metal complexes have good oxidation–reduction activities and chemical stabilities. The planar structure of a macrocycle increases the electron density of the central atom and improves the conductivity [16]. Therefore, most studies have employed iron phthalocyanine (iron (II) phthalocyanine, FePc) and cobalt tetramethoxyphenylporphyrin (CoTMPP) as catalysts. Zhao et al. coated carbon felt with FePc and CoTMPP as catalysts to produce a cathode electrode, and found that under a neutral pH, the current density increased from 0.40 to 0.97 mA/cm^2^, which proved that transition metallic macrocycles have good oxidation–reduction abilities [17]. Deng et al. employed a combination of CoTMPP and FePc as a macrocycle catalyst, and found that the power density with the Co/Fe/macrocycles was 751 mW/m^2^. This was 1.5 times the power density with the Pt/C catalyst under the same conditions, which showed that transition metallic macrocycles have excellent electrocatalytic activities [18].

Carbon nanotubes (CNTs) have properties such as a high specific surface area, high mechanical strength, and ductility, as well as excellent chemical stability and conductivity, which make them a promising pad material. Wang used Mo_2_C/CNTs as the catalyst when investigating the performance of a microbial fuel cell system. When using a carbon felt electrode modified by Mo_2_C/CNTs with 16.7 wt % Mo, the density of the power generated by the system was 1050 mW/m^2^, which was six times the density when employing only CNTs or Mo_2_C [19]. In a study by Ghasemi et al., a polymer polypyrrole/CNT composite material on carbon paper generated power at 113.5 mW/m^2^, which was higher than that with unmodified carbon paper (69.1 mW/m^2^) [20]. This proved that CNTs have good electrocatalytic performances in microbial fuel cells. In comparison with carbon nanotubes, it is found that carbon black particles easily cluster, which reduces the utilization rate of the platinum catalyst. In addition, the association of platinum and carbon black is unstable. Gradual separation may occur during the operation of the fuel cell system over a long period, which reduces the overall performance [21]. Shaijumon compared the performances of proton exchange membrane fuel cells (PEMFCs) using carbon black and CNTs as carriers, with the same weight percentage of platinum catalyst. The results showed that when the platinum catalyst was combined with the CNTs, the density of the current generated by the system was 535 mA/m^2^, which was higher than that obtained (310 mA/m^2^) when the platinum catalyst was loaded on carbon black. This proved that the platinum catalyst was distributed more uniformly on the surface of the CNTs, making their oxidation–reduction ability better than that of the catalyst obtained by combining carbon black and platinum [21].

In this research, it was expected that the utilization rate of the serialized CNT catalyst in a bio-electro-Fenton microbial fuel cell and the overall efficiency of the system could be improved using an iron phthalocyanine compound with aligned CNTs on the surface of a stainless steel (SS316) electrode.

## 2. Materials and Methods

In this research, we employed FePc/CNTs on an SS316 pad as the cathode electrode in a bio-electro-Fenton microbial fuel cell, and the overall electrical properties of the system and the dye decolorization were investigated.

### 2.1. Electrode Production

A 30 mm × 50 mm × 5 mm piece of carbon felt (CeTech Co., Ltd., Taichung, Taiwan) was employed as the electrode for the anode. Hydrogen peroxide and deionized water (in a 1:9 ratio) were poured into a beaker and heated to 90 °C, and a hydrophilic treatment was performed for 3 h. SS316 (Qunlong Steel Industry Co., Ltd., Yilan, Taiwan) was employed as the cathode electrode. The composition of SS316 is provided in Table 1. The electrode size was 20 mm × 30 mm × 2 mm. The impurities on the surface of the stainless steel were removed with various grades of sandpaper used in the following sequence: 100, 240, 400, and 600. The ground stainless steel electrode was then placed in an acetone solution, removed after 30 min of ultrasound cleaning, wiped, and dried.

### 2.2. The Fabricaiton Process of Composite FePc/CNT/SS316 Cathode

A uniform 0.5 mL drop of iron pentacarbonyl (Sigma-Aldrich, Saint Louis, MO, USA) on the bare SS316 plate was placed by pipet. The iron pentacarbonyl serves as the catalyst for CNT synthesis. Thermal pyrolysis chemical vapor deposition (TPCVD) was then employed to deposit CNT on the SS316 plate. Prior to the CNT synthesis process, the TPCVD furnace was heated and vacuumed to 750 °C and 50 mTorr, respectively, and followed by purging with nitrogen gas of 10 sccm. Then, the furnace was filled with acetylene gas of 50 sccm and/or argon gas of different flow rate, 100, 500, or 800 sccm, to start the CNT synthesis process. The samples were then naturally cooled to room temperature after the previous CNT synthesis process. A composite CNT/SS316 cathode electrode was completed at this stage. A stirred solution of 0.018 g iron phthalocyanine (FePc) and 1 mL tetrahydrofuran (THF) was then dropped on the composite CNT/SS316 cathode plate and subjected to ultrasonic oscillation for 30 min to form a uniform layer on the composite CNT/SS316 cathode plate. After baking in an oven of 80 °C for 24 h, the composite FePc/CNT/SS316 cathode electrode was completed.

### 2.3. Construction of Bio-Electro-Fenton Microbial Fuel Cell System

**Figure 1:**
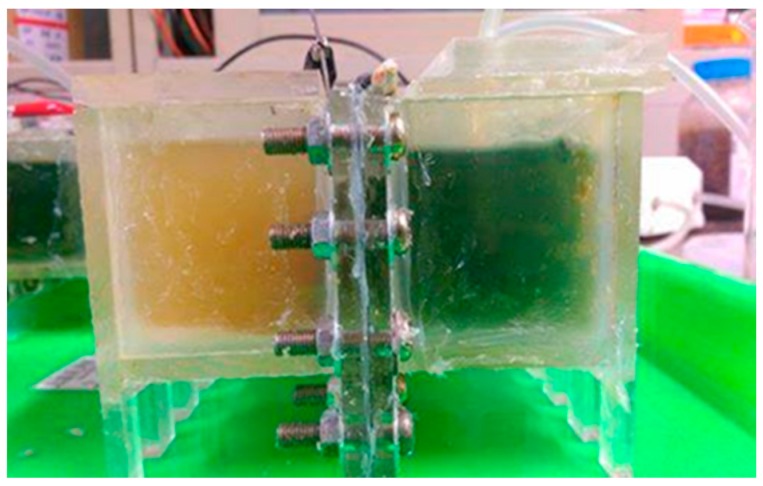
Experimental setup (left: anode chamber, right: cathode chamber).

The cathode and anode chambers of the double-chamber body employed in this system had a total capacity of 130 mL, with a DuPont™ Nafion^®^ PFSA N-117 (DuPont Limited, Wilmington, DE, USA) proton exchange membrane placed between them to perform the mass transfer reaction in the microbial fuel cell, as shown in Figure 1. The anode solution was wastewater from dairy products (Zhong Shumao Laboratory, Yilan, Taiwan), and the cathode solution was a 0.3 M iron (II) sulfate (FeSO_4_·7H_2_O, Japan Reagent, Tokyo, Japan) solution mixed with active carbon black dye at 1000 ppm.

### 2.4. Measurement of Electrical Quantities

In this study, the electrical quantities were measured using an electrochemical workstation (Zive SP1, WonATech, Seoul, Korea) and Zive SM (WonATech). The cathode and anode of the microbial fuel cell were connected to the electrochemical workstation to measure the voltage, current, and the cell polarization curve of the cell. The changes in these parameters were simultaneously recorded using a data acquisition system (Jiehan-5020, Jiehan Technology Corp., Taichung, Taiwan).

The constant current measurement was used to calculate the impedance loss within the microbial fuel cell by controlling the current, and the overall calculation of the cell polarization potential (E_cell_) is shown in Equation (7):

E_cell_ = E_n_ − η_ohm_ − η_act_ − η_conc_, (7)

Here, η_ohm_ is the ohmic polarization potential loss, η_act_ is the activation polarization potential loss, and η_conc_ is the concentration polarization potential loss.

To calculate the power density, the power (P) can be obtained from the relationship between the voltage (V_cell_) and the current (I), as shown in Equation (8). When an external load resistance (R_ext_) was added to the cell, the power (P) and current (I) could be obtained from the measured voltage (V_cell_) using Equations (9)–(11):

(8) $$P={\mathrm{IV}}_{\mathrm{cell}},$$

(9) $$I=V_{\mathrm{cell}}/R_{\mathrm{ext}},$$

(10) $$P=I^{2}\cdot R_{\mathrm{ext}},$$

(11) $$P=V_{\mathrm{cell}}^{2}/R_{\mathrm{ext}},$$

The power calculation was analyzed based on the polarization curve, with the system potential measured by controlling the current and calculating the power (P) output of the system using the current-voltage correlation. Cyclic voltammetry was conducted using three electrodes. The auxiliary electrode was made of platinum, and the reference was an Ag/AgCl electrode. The working electrode was placed at the location of the electrochemical reaction of the electrode, and the scanning rate was 10 mV.

The anolyte solution contained the mixed solution of dairy wastewater, including yogurt and milk. The anolyte solution contained microorganisms. We filled the anode chamber with half of the anolyte solution and half of glucose. Then we connected the anode electrode with a 1 kΩ resistor and monitored the voltage drop across the resistor. After a few hours, the voltage started to drop. Once it fell to approximately 5 mV, we replaced the anolyte solution completely. We repeated the above processes until the voltage reached at least 100 mV, at which point the microorganism incubation was complete. After that, we removed the resistor and started up the bio-electro-Fenton system for one-day discharging. The start-up process was completed at this stage. We reconnected the resistor and continued monitoring the voltage drop during the operation of the bio-electron-Fenton system. Once it fell to approximately 50 mV, we partially replaced the anolyte solution with glucose.

### 2.5. Preparation for Dye Decolorization

**Figure 2:**
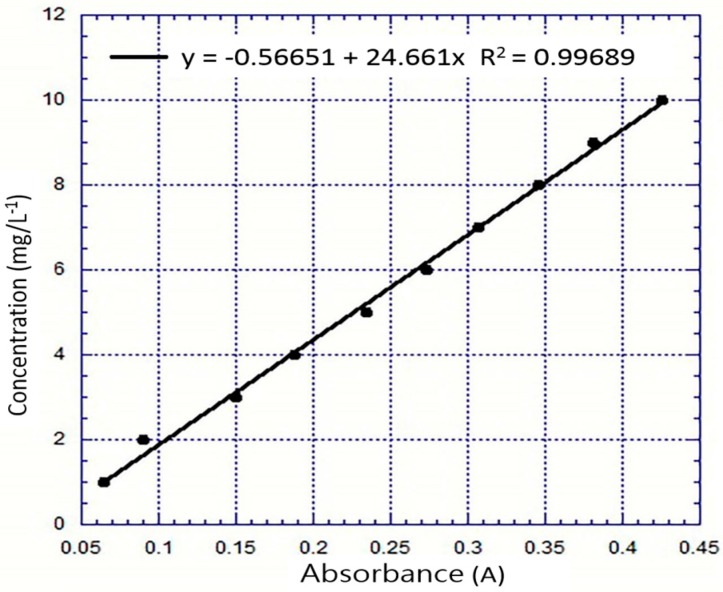
Plot of RB5 concentration vs. absorbance value in aqueous solution analyzed using a spectrophotometer.

The dye wastewater employed in the cathode chamber in this research was active carbon black (RB5). It had an absorption wavelength of 595 nm, a molecular formula of C_26_H_25_N_5_O_19_S_6_·4Na, and a molecular weight of 991.82. It contained a nitrogen-nitrogen double bond (N=N), which could be broken by the electron-Fenton reaction, resulting in a decolorization effect after the reduction of the chroma of RB5 within the cathode chamber. This proved that a continuous electron-Fenton reaction occurred. RB5 is a commonly used dye in the experimental detection of dye in wastewater. In the experiment, a comparison of the concentration and absorbance values of RB5 was performed using a spectrophotometer (SH-U880, Shishin Technology Corp., Taipei, Taiwan), and the variation of the RB5 concentration vs. absorbance is shown in Figure 2. The decolorization experiment was performed by (D) diluting 1.5 mL of electrolyte and 1.5 mL of distilled water in the sampling chamber and collecting samples on an hourly basis for 12 h. Before and after the experiment, the spectrophotometer was used to make comparisons between the visible light and the absorbance value, as shown in Equation (12):

(12) $$D=\frac{{\text{A}}_{\text{i}}-{\text{A}}_{\text{t}}}{{\text{A}}_{\text{i}}}\times 100\%,$$

A_i_ = Initial absorbance value, A_t_ = Measured absorbance value.

The experimental results of this research, including analyses of the cyclic voltammograms, analyses of the fixed resistance discharge, the electrochemical analysis, and the decolorization, were plotted with standard graphics software (Kaleida Graph, K-graph, Synergy Software), and the error in the values was in the range of ±5%.

## 3. Results and Discussion

### 3.1. Morphology of CNT Formation 

**Figure 3:**
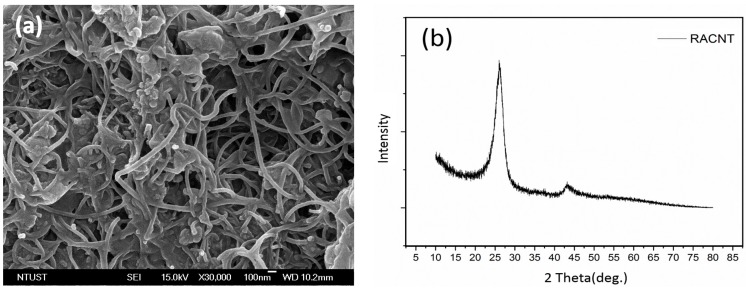
(**a**) The field emitted SEM images of carbon nanotubes is random alignment magnified 30,000 times; (**b**) XRD patterns of RACNT.

In the present work thermal pyrolysis chemical vapor deposition (TPCVD) to deposit CNT on the SS316 stainless steel plate was employed. The TPCVD furnace was heated and vacuumed to 750 °C and 50 mTorr, respectively, followed by purging with nitrogen gas at 10 sccm. Then, the furnace was filled with acetylene gas at 50 sccm to start the CNT deposition process for 20 min. Figure 3a shows the field-emitted SEM image, showing that the resulting CNT is randomly alignment, referred to as RACNT in the following text. The X-ray diffraction, Figure 3b, shows that two intensity absorption peaks occurred at 2θ of 26.0° and 43.0°, respectively, which were exactly the absorption peaks of CNT [22].

**Figure 4:**
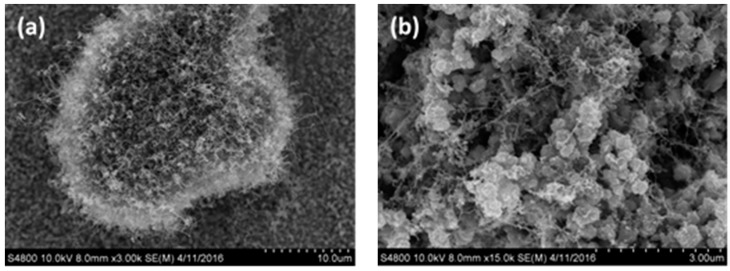
SEM images of carbon nanotubes prepared using argon gas at a flow rate of 100 sccm: (**a**) Magnified 3000 times; (**b**) Magnified 15,000 times.

Figure 4a shows the surface morphology of the CNTs generated on the surface of the stainless steel within 20 min, as observed using SEM, when the flow velocity of the argon gas was 100 sccm, the acetylene flow velocity was 10 sccm, and the temperature was 750 °C. This shows that there are agglomerates of CNTs on the stainless steel surface, with no CNTs generated in other forms. A SEM image at a magnification of 15,000 times is shown in Figure 4b, where it is seen that impurities of non-tubular carbon appear in the spaces between the CNT agglomerates. These impurities were generated because after the decomposition at high temperature, a portion of the carbon ions within the quartz tube clustered together, giving an amorphous carbon deposition in parallel with the generation of CNTs with the catalyst on the surface of the stainless steel, with particles stuck in the spaces between the nanotubes [23].

**Figure 5:**
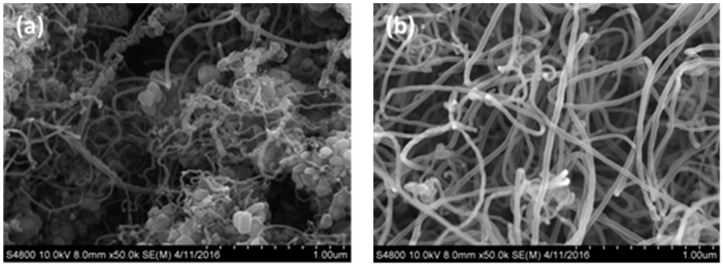
SEM graphs carbon nanotubes generated using argon gas flow velocity of 500 sccm: (**a**) Magnified 5000 times; (**b**) Magnified 50,000 times.

The morphology observed by SEM (Figure 5a) showed that CNTs with impurities were generated on the surface of the stainless steel electrode within 20 min when the argon gas flow rate was 500 sccm and the acetylene flow rate was 10 sccm at 750 °C. In addition to the CNTs and impurities, a sparse distribution of individual tubes was found. The agglomeration was relatively low, and disordered CNTs were formed, as shown in Figure 5b.

**Figure 6:**
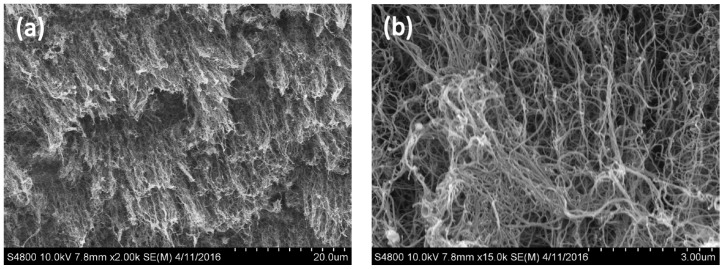
SEM images of carbon nanotubes formed using argon gas flow rate of 800 sccm: (**a**) Magnified 2000 times; (**b**) Magnified 15,000 times.

Figure 6a shows the morphological observation with SEM of the CNTs generated on the stainless steel surface within 20 min when the argon gas flow rate was 800 sccm and the acetylene flow rate was 10 sccm at 750 °C Figure 6. It is clearly seen that, with an argon flow rate of 800 sccm, the generated CNTs are widely distributed on the surface of the stainless steel without agglomeration. Moreover, it can be observed that the CNTs were generated in an upward direction. An image with a magnification of 15,000 times is shown in Figure 6b, and no impurity particles are seen in the interspaces between the nanotubes. The generated CNTs are arranged in an orderly way, with their tips slightly bent. This was because of the influence of gravity during their generation. Thus, the tops of the CNTs provided mutual support for each other.

The above results show that impurities may hinder the generation of CNTs [18], and the flow velocity of the argon gas has an absolute impact upon the generation of CNTs. When the argon gas flow rate was higher, the generated CNT structure had a lower probability of curling, along with aligned growth. This result showed that the CNTs were similar to those observed in previous research. When the CNTs were generated with an argon gas flow rate of 800 sccm and acetylene flow rate of 10 sccm, a high yield of CNTs could be observed [23]. In addition, the nanotubes showed an aligned generation phenomenon because of the lower concentration of impurities [9].

### 3.2. Analyses of Iron Phthalocyanine Component

**Figure 7:**
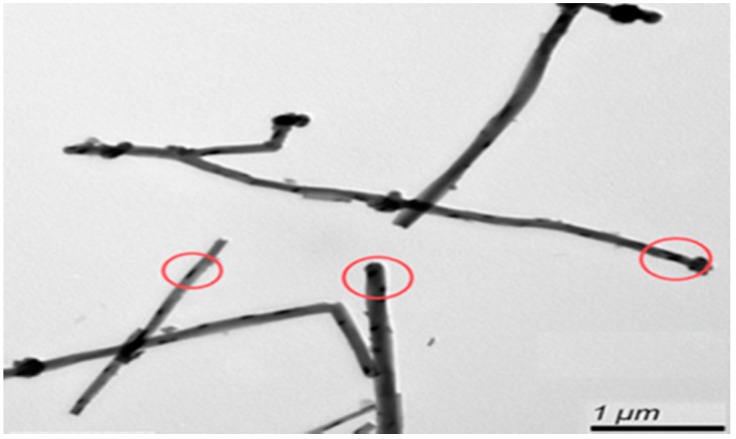
TEM image of iron phthalocyanine on the surface of carbon nanotubes.

TEM was used to observe the form of the CNTs and catalyst, as shown in Figure 7. Many black spots can be observed, which were uniformly distributed on the surface of the CNTs, as highlighted by the circles.

**Figure 8:**
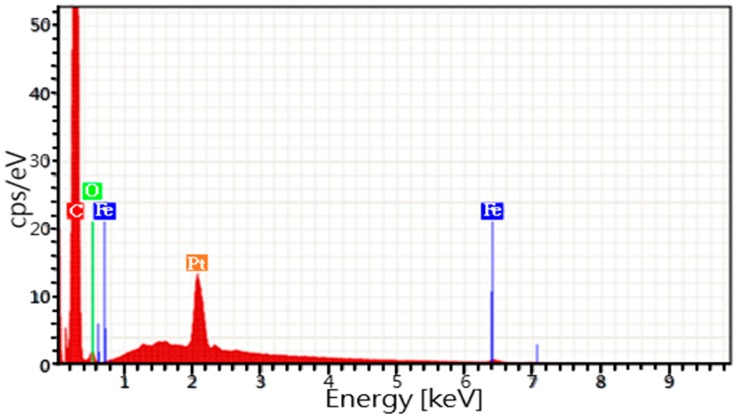
EDS analysis results for the carbon nanotube-iron phthalocyanine composite.

An EDS analysis of the CNT component was conducted, and the results are shown in Figure 8. The CNTs contain four elements (C, O, Pt, and Fe), among which carbon is the majority element, with the highest peak. The oxygen peak is due to the oxygen present in the instrument cavity, and a Pt coating was used to facilitate observation with the electron microscope. The trace iron element originated from the added iron phthalocyanine.

### 3.3. Cyclic Voltammetry Analyses

**Figure 9:**
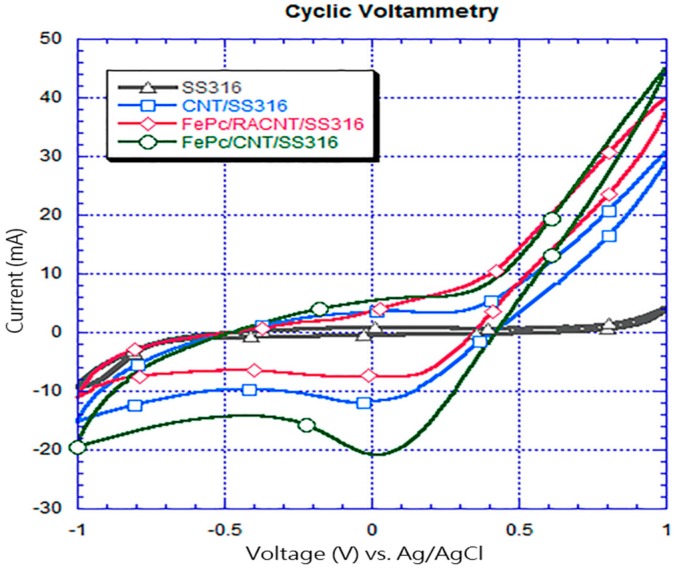
Measurement chart of cyclic voltammetry current-voltage.

The electrochemical activity generated by the electrode in the cell system was characterized using the cyclic voltammetry method. When the redox activity of the electrode was stronger, the measured peak was more pronounced. In this research, cyclic voltammetry analyses of four electrodes were performed at a scanning rate of 10 mV, and the results are shown in Figure 9. The reduction peak observed on the SS316 electrode is at the position with a reference voltage of 0.035 V and current of −0.37 mA. The reduction peak for the CNT/SS316 electrode was less than that of the stainless steel electrode, and the position of the reduction peak was at a voltage of −0.047 V and current of −12 mA. The position of the reduction peak of the FePc/RACNT/SS316 electrode (where RACNT represents random carbon nanotube) is at a voltage of −0.022 V and current of −8.6 mA. The reduction peak of the FePC/CNT/SS316 electrode with the added iron phthalocyanine is at a voltage of 0.018 V and current of −21 mA. The reduction peaks observed in the four samples were near the reference potential of 0 V, and no oxidation peak reaction was observed because the electrodic reaction was irreversible. The addition of CNTs could improve the electrochemical activity of the electrode, and iron phthalocyanine was a good catalyst, as shown in the studies by Deng et al. [18] and Pacios et al. [24], because it improved the electrochemical activity. In this research, CNTs and iron phthalocyanine were combined on the SS316 electrode as the catalyst, which could greatly improve the electrochemical activity of the stainless steel electrode.

In the study of the effect of the generation direction of the CNTs on the electrochemical activity, the observations shown in Figure 9 indicate that the reduction peaks generated by the SS316 electrode for different argon gas flows were not significant, and the measured electrochemical activity was obviously worse than that for the CNT/SS316 electrode. Although the reduction peak could be clearly observed for the FePc/RACNT/SS316 after the FePc was added, it was not better than the CNT/SS316 electrochemical activity generated with aligned nanotubes. In addition, it can be observed that the FePc/CNT/SS316 electrode has a reduction peak and the best electrochemical activity. Compared with the results discussed in Section 3.1 for an argon gas flow velocity of 800 sccm, the direction of the generated CNTs was easily discerned. The excessive impurities on the CNTs generated with different argon gas flow rates are attached to the CNTs, which may affect their surface area and generation direction [25,26]. Thus, they would result in a low redox activity measured for the randomly-oriented CNTs.

### 3.4. Analyses of Discharge with Constant Resistance

**Figure 10:**
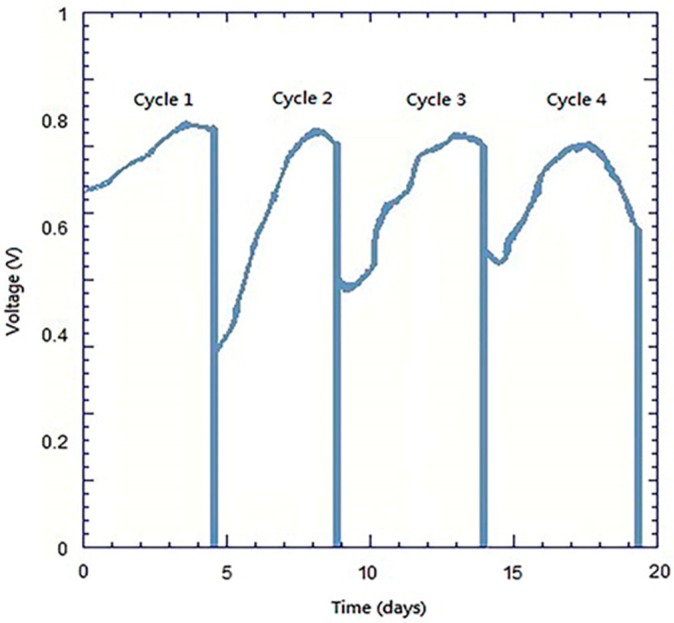
Plot of discharge voltage vs. time for FePc/CNT/SS316.

In Figure 10 the long-term discharge of the present bio-electro-Fenton system employing the FePc/CNT/SS316 composite cathode is reported. We conducted four discharge cycles, and each cycle lasts about four days. Each discharge cycle begins at the start-up process mentioned at the end of the Section 2.4 and ends when reaching a maximum output voltage. Then, the chambers are cleaned up new anolyte and catholyte solutions are added, and the next discharge cycle is started. Figure 10 shows that each discharge cycle can reach a stable maximum voltage level of 0.8 V, which verifies the reproducibility of the present bio-electro-Fenton system employing the composite FePc/CNT/SS316 cathode. Additionally, the maximum output voltage (0.8 V) agrees with all MFCs’ open circuit voltages (0.7–0.8 V) mentioned by Ren [9]. In summary, the long-term discharge proves that the composite FePc/CNT/SS316 is suitable for being the cathode of a bio-electro-Fenton system.

### 3.5. Analysis of Electrical Quantities

**Figure 11:**
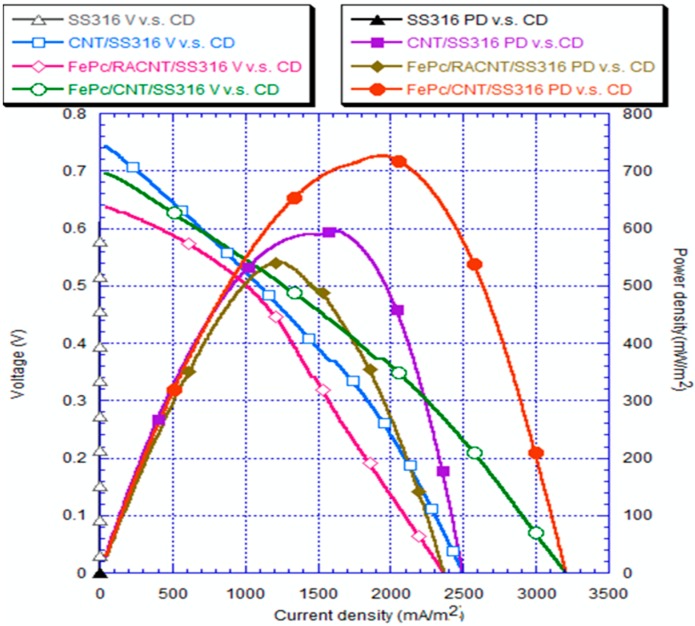
Plot showing comparison of electrical properties of voltage, current, and power on the fifth day after initiation of the bio-electro-Fenton system.

The electrical quantity of a bio-eletron-Fenton system is characterized by the three physical quantities: open-circuit voltage, current density, and power density. The anode electrode of the present bio-electron-Fenton system is carbon felt, while its cathode electrode is made of a SS316 stainless steel on which CNT and FePc are deposited sequentially. Since carbon felt was commonly employed as the electrode of microbial fuel cells in many literatures, it is not within the investigation scope of the present work. The present work is the first that employs the composite of FePc/CNT/SS316 as a cathode electrode. Here the influence of the FePc/CNT/SS316 cathode on the electrical properties of a bio-electro-Fenton system is discussed. Figure 11 compares the electrical properties of a bio-electro-Fenton system employing four different treatments of cathode electrodes, bare SS316, SS316 covered by aligned CNT, SS316 covered by FePc and random CNT, and SS316 covered by FePc and aligned CNT. Its abscissa is current density, the left-ordinate is voltage, and the right-ordinate is power density. The I–V curves (V vs. CD) refer to the left-ordinate, while the power curves (PD vs. CD) refer to the right-ordinate. For the case of bare SS316, its open-circuit voltage (the intersection point of I–V curve and left-ordinate) is 0.6 V, the maximum current density is 3.42 mA/m^2^ (the intersection point of I–V curve and abscissa), which is too tiny to read in this figure, and the maximum power density is 0.29 mW/m^2^. For the case of CNT/SS316, its open-circuit voltage is 0.74 V, the maximum current density is 2497 mA/m^2^, and the maximum power density is 588.96 mW/m^2^. For the case of FePc/RACNT/SS316, its open-circuit voltage is 0.64 V, the maximum current density is 2365 mA/m^2^, and the maximum power density is 540.44 mW/m^2^. For the case of FePc/CNT/SS316, its open-circuit voltage is 0.7 V, the maximum current density is 3206 mA/m^2^, and the maximum power density is 726.55 mW/m^2^.

Bare SS316 shows the worst electrical quantity, both of I–V and power density curves, which is due to its poor electron transport property [27]. CNT/SS316 shows better electrical properties than that of SS316, which reveals that CNT can promote the electron transport in stainless steel efficiently [18]. FePc/CNT/SS316 shows further better electrical properties than that of CNT/SS316, which is due to FePc being a catalyst and its planar structure can easily adhere to CNT to promote the reduction reaction at the cathode [17,28]. It should be mentioned here that the direction of the CNTs are aligned by tuning the flow rate of argon during the chemical vapor deposition process of CNTs. To verify the influence of the alignment of CNTs, another case of random CNT, FePc/RACNT/SS316, is compared here again. As shown in Figure 11, FePc/RACNT/SS316 reveals worse electrical properties than those of CNT/SS316 and FePc/CNT/SS316. This is because the RACNT were intertwined, which leads to a lower specific surface area than that of aligned CNTs. Low specific surface area limits the utilization of the catalyst FePc [29]. In summary, CNTs can effectively promote the conductivity of SS316 stainless steel, the catalyst FePc can easily adhere to CNTs to promote the reduction reaction at the cathode, and the alignment of CNTs is critical to the utilization of FePc due to its high specific surface area. Zagal et al. [16] employed a composite nano-Mo_2_C/CNT/carbon-felt as the anode electrode of a microbial fuel cell. The present work reaches a maximum open-circuit voltage 0.74 V which is slightly higher than that of it (0.702 V). Our maximum current density reaches about 3.2 A/m^2^, which is slightly lower than that of Zagal et al.’s electrode (11.2 A/m^2^). Our maximum power density reaches about 0.73 W/m^2^ which is a little lower than that of Zagal et al.’s electrode (1.05 W/m^2^). The performance of electricity generation in the present work is slightly lower than that of [16]. However, the present work is a bio-electro-Fenton system whose electricity generation is to support the Fenton reaction at the cathode. The performance of the Fenton reaction is characterized by the dye declorization at the cathode, which will be discussed in the Section 3.6.

### 3.6. Decolorization Analyses 

**Figure 12:**
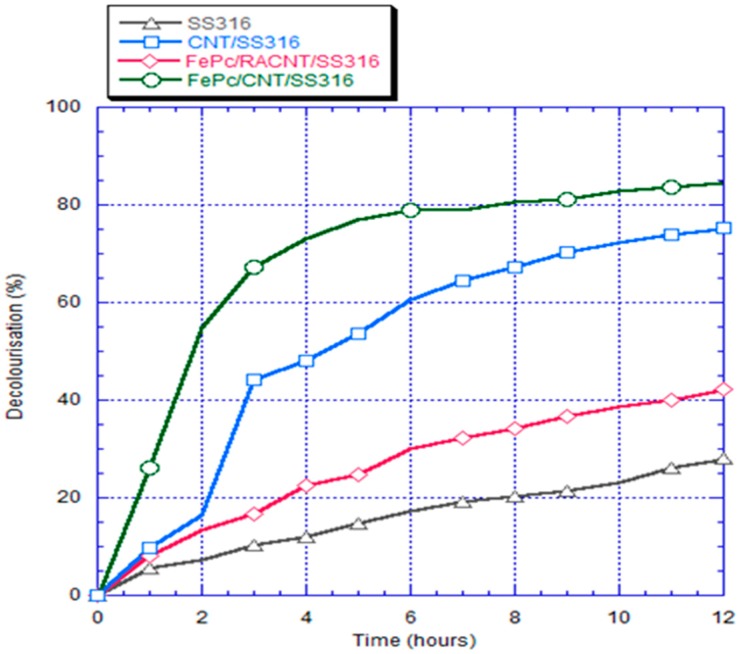
Plot showing rates of decolorization for different electrode systems (concentration of RB5: 50 ppm).

The measurement results for the decolorization of the RB5 dye within 12 h using the different cathode electrodes are shown in Figure 12. In these figures, the decolorization achieved with the FePc/CNT/SS316 electrode is 84.6%, which is the maximum degree of decolorization among the four cathode electrodes. The electrodes can be listed in the order of decreasing decolorization activity as follows: the CNT/SS316 electrode, the FePc/RACNT/SS316 electrode, and the SS316 electrode, which had decolorization rates of 74.0%, 42.1%, and 28%, respectively. The color of RB5 dye has different chromaphore and concentration values due to the functional group, and the electron-Fenton reaction breaks the bond of the functional group with ·OH, resulting in the reduction of the chroma of the dye. In the bio-electro-Fenton system, the maximum number of hydroxyl radicals was generated when FePc/CNT/SS316 was used as the cathode electrode. Thus, the most significant decolorization of the RB5 dye was achieved with this electrode. In terms of the electrical properties of the bio-electro-Fenton microbial fuel cell system, the current density generated by the system had a close relationship with the decolorization rate of the RB5 dye. When this electrical property was improved, the relative demand for hydroxyl radicals was high, and the decolorization of the RB5 dye was fast. The decolorization effect with the FePc/CNT/SS316 electrode, which had the maximum current density, was the best. This result showed that the advantages and disadvantages of the electrical properties of the bio-electro-Fenton microbial fuel cell system were related to the degree of dye decolorization.

**Figure 13:**
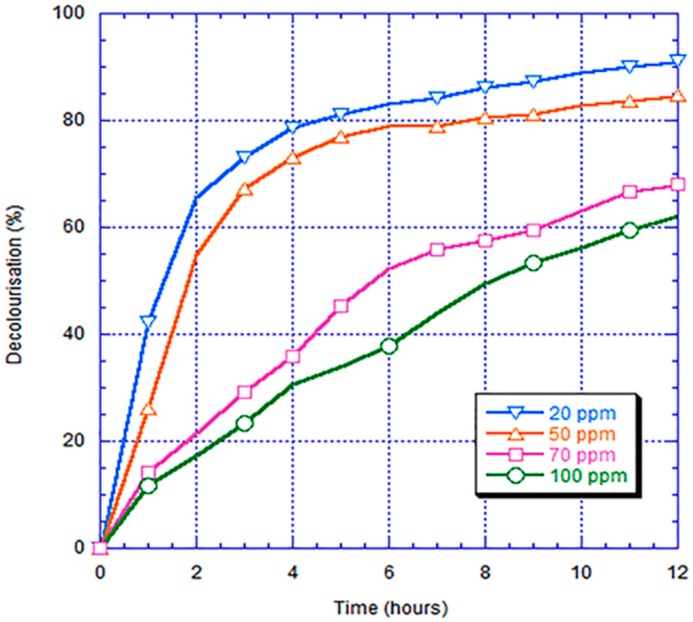
Chart of absorbance value vs. time for different concentrations of RB5.

The decolorization rates for the bio-electro-Fenton microbial fuel cell with the FePc/CNT/SS316 electrode and different RB5 concentrations of 20, 50, 70, and 100 ppm are shown in Figure 13. These results show that the decolorization degree had a boundary around the concentration of 50 ppm. When the concentration was greater than 50 ppm, the decolorization degree within 12 h was approximately 60%–70%. When the concentration was less than 50 ppm, the decolorization degree could reach more than 80%. Thus, when the RB5 concentration was higher, the time required for the decolorization by the bio-electro-Fenton system was longer.

## 4. Conclusions

In this research, the electrical properties of bio-electro-Fenton microbial fuel cells with FePc/CNT/SS316 electrode were investigated, and the conclusions are as follows.

1. CNTs were fabricated using TPCVD, and it was found that the argon gas flow velocity had an absolute impact upon the generation of CNTs. When the argon gas flow velocity was higher, the probability of curling in the generated CNT structure was lower, with aligned generation. The CNTs were generated with good alignment under an argon gas flow rate of 800 sccm and acetylene flow rate of 10 sccm.
2. In the measurements using the cyclic voltammetry method, the measured position of the reduction peak of the SS316 electrode had values of 0.035 V and −0.37 mA; the position of the reduction peak of CNT/SS316 had values of −0.047 V and −12 mA; the position of the reduction peak of the FePc/RACNT/SS316 electrode had values of −0.022 V and −8.6 mA; and the position of the reduction peak of FePc/CNT/SS316 had values of 0.018 V and −21 mA. This showed that the iron phthalocyanine and serialized CNT catalysts were conducive to improving the redox activity of the electrode, while the FePc/CNT/SS316 electrode could improve the redox activity of the SS316 electrode.
3. The current density and power density of the system with the FePc/CNT/SS316 electrode were 3206.30 mA/m^2^ and 726.55 mW/m^2^, respectively; while the current density and power density generated by the SS316 electrode were 3.42 mA/m^2^ and 0.28 mW/m^2^, respectively. These results showed that the current density and power density of the modified FePc/CNT/SS316 electrode were 937 and 2594 times higher than those of the system with only the SS316 electrode. In addition, in the experiment on the fixed resistance discharge of the bio-electro-Fenton microbial fuel cells, after exchanging the chamber solution over four cycles, the FePc/CNT/SS316 electrode could still reach a stable voltage output of 0.8 V.
4. In the decolorization experiment, the electricity generated by the anode chamber of the bio-electro-Fenton microbial fuel cell system could independently promote the initiation of the electron-Fenton system reaction of the cathode chamber. The decolorization rates of the SS316 electrode, FePc/RACNT/SS316 electrode, CNT/SS316 electrode, and FePc/CNT/SS316 electrode within 12 h were 28%, 42.1%, 74%, and 84.6%, respectively, among which the decolorization efficiency of the modified FePc/CNT/SS316 electrode was the best.
5. In this research on a bio-electro-Fenton microbial fuel cell system, the anode was employed for electricity generation and the cathode was employed for the Fenton reaction, achieving the effects of energy generation and sewage disposal at the same time. In addition, an iron phthalocyanine and serialized CNT composite was adopted as the catalyst, which could improve the electrical properties and decolorization effect, and has great potential for improving bio-electro-Fenton microbial fuel cells in the future.

## Figures and Tables

**Table 1:** Analysis results for SS316 components.

Component	Si	Mo	Cr	Mn	Fe	Ni
Content (%)	0.86	2.23	17.78	1.09	67.41	10.63
